# Solidified floating organic droplet microextraction coupled with HPLC for rapid determination of trans, trans muconic acid in benzene biomonitoring

**DOI:** 10.1038/s41598-021-95174-5

**Published:** 2021-08-03

**Authors:** Fatemeh Dehghani, Fariborz Omidi, Omidreza Heravizadeh, Saeed Yousefinejad

**Affiliations:** 1 Student research committee, Shiraz University of Medical Sciences , Shiraz, Iran; 2 Research Center for Environmental Determinants of Health (RCEDH), Health Institute, Kermanshah University of Medical Sciences, Kermanshah, Iran; 3 Division of Metabolomics, Medical Institute of Bioregulation, Kyushu University, Fukuoka, 812-8582 Japan; 4 Department of System Life Sciences, Graduate School of Systems Life Sciences, Kyushu University, Fukuoka, 819-0395 Japan; 5 Research Center for Health Sciences, Institute of Health, Department of Occupational Health Engineering , School of Health, Shiraz University of Medical Sciences, 71645-111 Shiraz, Iran

**Keywords:** Chemistry, Analytical chemistry

## Abstract

Benzene is one of the carcinogenic compounds in the work environments. Exposure assessment of benzene through biological monitoring is an acceptable way to accurately measure the real exposure in order to conducting the health risk assessment, but it is always complicated, laborious, time consuming and costly process. A new sensitive, simple, fast and environmental friendly method was developed for the determination of urinary metabolite of benzene, trans trans muconic acid (t,t-MA) by dispersive liquid–liquid micro extraction based on solidification of floating organic droplet coupled with high-performance liquid chromatography with ultra violet detector. Central composite design methodology was utilized to evaluate the effective factors on the extraction output of the target metabolite. The calibration curve was plotted in the concentration ranges of 0.02–5 µg mL^−1^. The precision and accuracy of the method were assayed via the relative standard deviation (RSD%) and relative recovery (RR%) using spiked samples with three replications. The RR% and RSD% of the optimized method were 86.9–91.3% and 4.3–6.3% respectively. The limit of detection (LOD) of the method was 0.006 µg mL^−1^. The level of t,t-MA in real samples was ranged from 0.54 to 1.64 mg/g creatinine. We demonstrated that t,t-MA can be extracted and determined by an inexpensive, simple and fast method.

## Introduction

Nowadays, organic solvents are extensively used in various processes because of their unique characters. Benzene, a well-known organic solvent, is applied to synthesize new material in different processes such as petrochemical, paint, adhesive process, rubber, and plastic^1^. Breathing and skin contact are regarded as the major paths of exposure to benzene^2^. The carcinogenicity of benzene (class I carcinogen) has been confirmed by the US Environmental Protection Agency (EPA) and International Agency for Research on Cancer (IARC)^3,4^. Many adverse health problems may be created through benzene intake. The effect of benzene on the hematopoietic organ is one of the main adverse health effects that has long been considered. Suppression of blood factors such as hemoglobin and white blood cells has been reported in people exposed to benzene^5,6^. Moreover, long-term exposure may suppress the immune system and reduce the number of lymphocytes^7^.

There are two ways to accurately determine the level of exposure to pollutants: air sampling and biological monitoring. Benzene could enter the body via inhalation, gastrointestinal system and skin contact^8^. Therefore, biological monitoring is necessary for precise determination of real exposure to benzene. During metabolism process, benzene is metabolized to different ring-opened and hydroxylated compounds such as trans, trans-muconic acid (t,t-MA), phenol, catechol and S-phenylmercapturic acid^9,10^. Phenol and catechol metabolites were associated with high concentration of benzene exposure (higher than 10 ppm). However, in low levels of benzene (lower than 1 ppm), no association was found between urinary levels of phenol and catechol compounds^11^. It has been found that there is a good correlation between urinary t,t-MA and concentration of benzene in air and blood in low levels of benzene exposure^12,13^. Furthermore, the American Conference of Governmental Industrial Hygienists (ACGIH) has introduced t,t-MA as a urinary biomarker of benzene exposure^14^.

Oxidation is the first step of benzene metabolism which produces benzene oxide. Under the influence of the metabolism processes, benzene oxide undergoes various fates. As an alternative way during the metabolism process, benzene ring may be opened in the benzene oxide or oxepin step via the reactive intermediate muconaldehyde. By oxidizing the trans,trans-mucondialdehyde, t,t-MA is produced^15^.

Although monitoring of biological media is an acceptable way to accurately measure the real exposure to the compounds, it is always complicated, laborious, time consuming and costly process^16^. The complexity of the biological fluids and also low amounts of the metabolites are the major barriers for analyzing the biomarkers in theses media. Sample preparation is a good solution to conquer these problems.

Up to now, various modern, high-throughput, miniaturized sample preparation techniques coupled with analytical instruments have been introduced for trace monitoring of t,t-MA in urine samples^17–19^. Due to low organic solvent consumption and high preconcentration factor, dispersive liquid–liquid microextraction (DLLME) and dispersive solid-phase microextraction (SPME) methods are among the most popular methods. The SMPE procedure requires preparation of the solid sorbent and subsequently analysis of the prepared sorbent by analytical instrument, which is time consuming and costly. The DLLME procedures are easy, rapid, and also require no extra characterization^20,21^. However, the use of toxic solvents (solvents used as extractant and disperser) is the main drawback of this method. Halogenated hydrocarbons are an example of these solvents, which are toxic and heavier than water^22,23^. As an attempt to solve these limitations, DLLME using a floating organic droplet (DLLME-SFOD) has been introduced with remarkable advantages over conventional extraction methods. The use of less toxic organic solvent, use of low organic solvent, operator safety, rapid extraction, and also environmentally friendly are some advantages of the proposed sample preparation compared to conventional DLLME^24^. In DLLME-SFOD technique, the density of the applied organic solvent is often lower than water, which help to be floated in the sample surface and simply collected from the sample media^25^. In this work, for the first time, a new DLLME-SFOD technique was optimized for the trace monitoring of t,t-MA from urine specimens. The applied organic solvent during solidification procedure has good consistency for the extraction of t,t-MA with excellent recovery which was comparable with routinely applied SPE procedure in samples collected from real exposed workers with benzene. Another important point in the current work is using response surface methodology for optimization of the solidified floating organic droplet microextraction in a complex sample such as urine. Here, we tried to show the importance of multivariate-optimization (instead of one-at-a-time process), to handle a multi-factor method such as SFOD especially for a complex real media. We think the obtained wonderful results can be related to this precise optimization and considering all factors and between-factors interactions.

## Experimental

### Reagents and solutions

t,t-MA (analytical standard) was obtained from Sigma–Aldrich (Darmstadt, Germany). 1-undecanol (99%), 1-dodecanol (99%), sodium hydroxide, sodium chloride, hydrochloric acid (HCl), acetic acid, and methanol (HPLC grade) were all provided from Merck Company (Darmstadt, Germany). Doubled distilled water was obtained from a milli-Q system (Bedford, MA, USA). Standard solutions of t,t-MA (1000 mg L^−1^) were prepared individually using methanol and double-distilled water at 1:4 ratio. The required working standards were daily prepared from the stock solution. The prepared stock solutions were kept at refrigerator and were freshly prepared after 10 days.

### Instrumentation

Chromatographic analyses were done by a HPLC system (HPLC, Knauer,Smartline system 1000, Berlin, Germany) combined with an ultraviolet detector (Knauer, 2000). The separation of analyte was performed via a C18 analytical column (Knauer, Eurospher 100–5 C18, 150 mm × 4.6 mm). A mixture of acetic acid 1%-methanol (70/30 v/v) with a flow rate of 1.0 ml/min was used as the mobile phase. The wavelength of UV detector was set at 274 nm. The injection was done manually using a 20µL stainless steel loop. A Metrohm 827 pH-meter (Metrohm, Switzer-land) was used for measurement of pH. Moreover, Hettich EBA 20 centrifuge was used for the separation of organic solvent from sample solution.

### *DLLME-SFO* procedure

**Figure 1:**
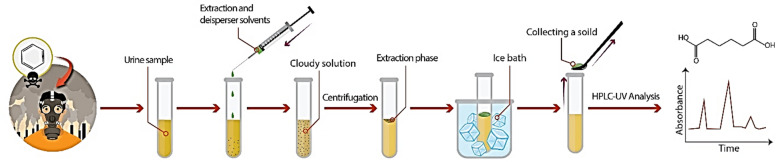
A schematic diagram of the utilized DLLME-SDOD procedure.

Figure 1 presents the schematic diagram of DLLME-SFOD procedure. In the proposed extraction method, a 10 mL of aqueous sample solution consist of 1.0 μg mL^−1^ of t,t-MA was poured into a 15-mL screw-cap glass centrifuge tube. pH of the solution was regulated in definite value (***A***) by stepwise addition of hydrochloric acid and sodium hydroxide solution. Then, the salt amount of the solution was adjusted using required percent (w/v) of NaCl (***D,***%). In the next step, a mixed solution of 1-undecanol, as extractant solvent (***B,*** µL) and the needed volume of methanol, as dispersive solvent, (***C,*** µL) was quickly introduced into the sample solution and the resultant mixture was agitated for 30 s using a vortex mixer. In this step, a cloudy suspension was created because of the dispersion of tiny droplets of 1-undecanol in the sample solution and subsequently, the extraction of analyte into 1-undecanol occurred quickly. The formed emulsion was then centrifuged for 5.0 min at 4000 rpm. After centrifugation, the tiny droplets of extractant solvent ^1-undecanol^ containing analyte were floated at the top of the sample solution due to the difference between the density of 1-undecanol and aqueous solution. The glass tube was then put into cold-water immersion for 5 min until the solidification of organic solvent occurred. Finally, the solidified organic droplet was put into a HPLC vial and brought to lab temperature. In lab temperature, the solidified droplet was quickly melted and then analyzed by the HPLC–UV.

### Real sample collection

Metabolite-free urine specimen was obtained from a healthy male in the analytical lab. Moreover, five end-shift urine samples from exposed workers of a petrochemical company were taken for monitoring of t,t-MA. All collected urine samples were stored at – 20 °C, prior to analysis. Sampling urine sample and human participation was done in compliance with the relevant regulations and the ethical principles outlined in the Helsinki’s declaration. This study was approved by the Ethics Committee of the Shiraz University of Medical Sciences.

### Sample treatment

Before analysis, both blank (samples taken from unexposed healthy male) and real samples (samples taken from workers who occupationally expose to benzene) were prepared according to the following method; 10 mL of each sample was transferred to a conical centrifuged tube and centrifuged for 10 min at 4000 rpm**.** The supernatant liquid was then filtered via a 0.45 μm filter and 5 mL of the obtained solution was poured to a vial. For reducing the effect of matrix, the urine sample was diluted to 10 mL using double distilled water. The obtained solution was then pre-concentrated using DLLME-SFOD procedure and finally analyzed by HPLC–UV.

### Experimental design

Different parameters including the solution pH, salt amount, type and volume of the disperser and extractant solvents, and extraction time affect the efficiency of the microextraction techniques. Therefore, an essential step in extraction techniques is determination of the optimum conditions of experiments. Experimental design methodology is an effective way for optimizing of the effective parameters on the extraction process. Saving time, enhancing the efficiency of the process, investigation of the interactions between parameters, and decreasing the errors with the minimum numbers of runs are some advantages of the experimental design method^26–28^. Response surface methodology (RSM) have been extensively utilized to link polynomial models with experimental data using different approaches, including central composite design (CCD). In this study, a CCD with five variables of pH (*A*), extractant solvent volume (*B,* μL), disperser solvent volume (*C,* μL), salt amount (*D* w/v,%)), and extraction time (min) (*E*) at five boundary levels (− α, − 1, 0, + 1, + α) was used (Table 1). The total runs number (N) (${\text{N}} = \frac{1}{2}$ 2^ K^ + 2 K + N_0_, which, k is introduced as the number of variables and N_0_ is the number of central points) was calculated to be 32 runs with central and axial points (α = 2) (Table S1, Supplementary information). The results obtained from the CCD were fitted to the following polynomial equation:

1 $$Y = \mathop \sum \limits_{i = 1}^{n} b_{i} x_{i} + \mathop \sum \limits_{i = 1}^{n} b_{ii} x_{i}^{2} + \mathop \sum \limits_{i < j}^{n} b_{ij} x_{i} x_{j} + \varepsilon$$

where Y is a predicted response, n is the number of parameters, xi, xj are independent variables in coded units ,b0, bi, bii, bij are the regression coefficients, and $\varepsilon$ is the residual error^29,30^. As denoted previously, we had five independent variables (i = 1,2,3,4,5) which is shown by *A,B,C,D, E* as code names. Subsequently, the analysis of variance (ANOVA) was used to determine the effects of linear, quadratic and interaction regression coefficient using Design Expert 7.1.3 software (Stat-Ease Inc., Minneapolis, Minnesota, USA). The adaptability between polynomial equation and response was evaluated using determination coefficient (R^2^). The significance of the polynomial equation terms was analyzed statistically by computing F value at *P* < 0.1. Because of high numerical values of peak area, and to have ordered results and plots, the peak areas was normalized using the maximum (highest peak area) and the normalized responses was utilized for further modeling, as shown in Table S1 (Supplementary information).

**Table 1:** The matrix of central composite design and responses.

Variables	Level			Star points(α = 2.0)	
	Low (− 1)	Central(0)	High (+ 1)	− α	+ α
A: pH	3	5	7	1	9
B:Extractant solvent volume (μL)	30	40	50	20	60
C: Disperser solvent volume (μL)	100	200	300	0	400
D: Salt amount (w/v,%)	2	4	6	0	8
E: Extraction time (min)	2	3	4	1	5

## Results and discussion

In this study, the DLLME-SFOD followed by HPLC–UV was optimized for the extraction and determination of t,t-MA from urine samples.

### Selection of the extractant and disperser solvents

In the DLLME-SFOD method, the choice of the suitable extractant and disperser solvents is critical to attain maximum extraction output. The extractant solvent was selected regarding some requirements such as high affinity to the analyte, low density (in compared to water), immiscible with water, high extraction output for target compound, low toxicity, compatibility with HPLC chromatographic separation, inexpensive, and low volatility. Among the mentioned requirements, density parameter is one of the most important parameters compared to others due to easy collection of the extractant solvent. Considering the above-mentioned requirements, some solvents such as 1-undecanol, 1-dodecanol and 2-dodecanol, as the most popular solvents, were candidate as the extractant solvents. Herein, because of practical ease, lowest cost and availability, 1-undecanol and 1-dodecanol, which had been introduced as the most efficient extractant solvent in previous studies^31,32^ were tested. The results showed that 1-undecanol was the best solvent and selected for additional examinations. Miscibility in both aqueous media and extractant solvent is the only requirement for selecting disperser solvent. In this work, acetone, acetonitrile and methanol were tested and methanol was chosen as the disperser solvent because of its ability to form a suspension with fine droplets in both media and significant potential for procedure in compare with other solvents such as acetonitrile and acetone during pre-tests.

### RSM-CCD

To prevent the impact of uncontrolled variables, the order of the experimental runs, shown in supplementary material, was done randomly in the CCD matrix. According to the outputs of ANOVA analysis on the collected data, the quadratic polynomial model was suitably fitted to the obtained data. Then to provide a refined model and to remove the factors or interaction terms with not-significant *P*-value (> 0.1) a backward elimination variable selection was done^33^ and the final findings as following equation:

2 $$\begin{aligned} {\text{Peak}}\;{\text{Area}} = & {364}.{27} - {32}.{24} \times {\text{A}} + 0.{38} \times {\text{B}} - 0.{53} \times {\text{C}} - {3}.{34} \times {\text{D}} \\ & \quad {-}{149}.{28} \times {\text{E}} + 0.0{4} \times {\text{AC}} + {7}.{39} \times {\text{AE}} + 0.0{2} \times {\text{CD}} + 0.{1}0 \times {\text{CE}} + {16}.0{6} \times {\text{E}}^{{2}} . \\ \end{aligned}$$

To evaluate the significance of the above multiple linear regression (MLR) model, Fisher’s statistical test (F-test) was used^33,34^. The F-value of the above-developed model was 27.23 and was higher than the critical F-value in required degree of freedom and shows the significance of that the model which is shown in Eq. (2). Another important criterion for confirming the validity of such MLR models, obtained from the table of experimental design, is a non-significant ‘Lack of Fit (LOF). F-value’ of the LOF in the current model was 0.669 and confirms that LOF was not significant, and represents the absence of pure error in the suggested MLR model.

**Figure 2:**
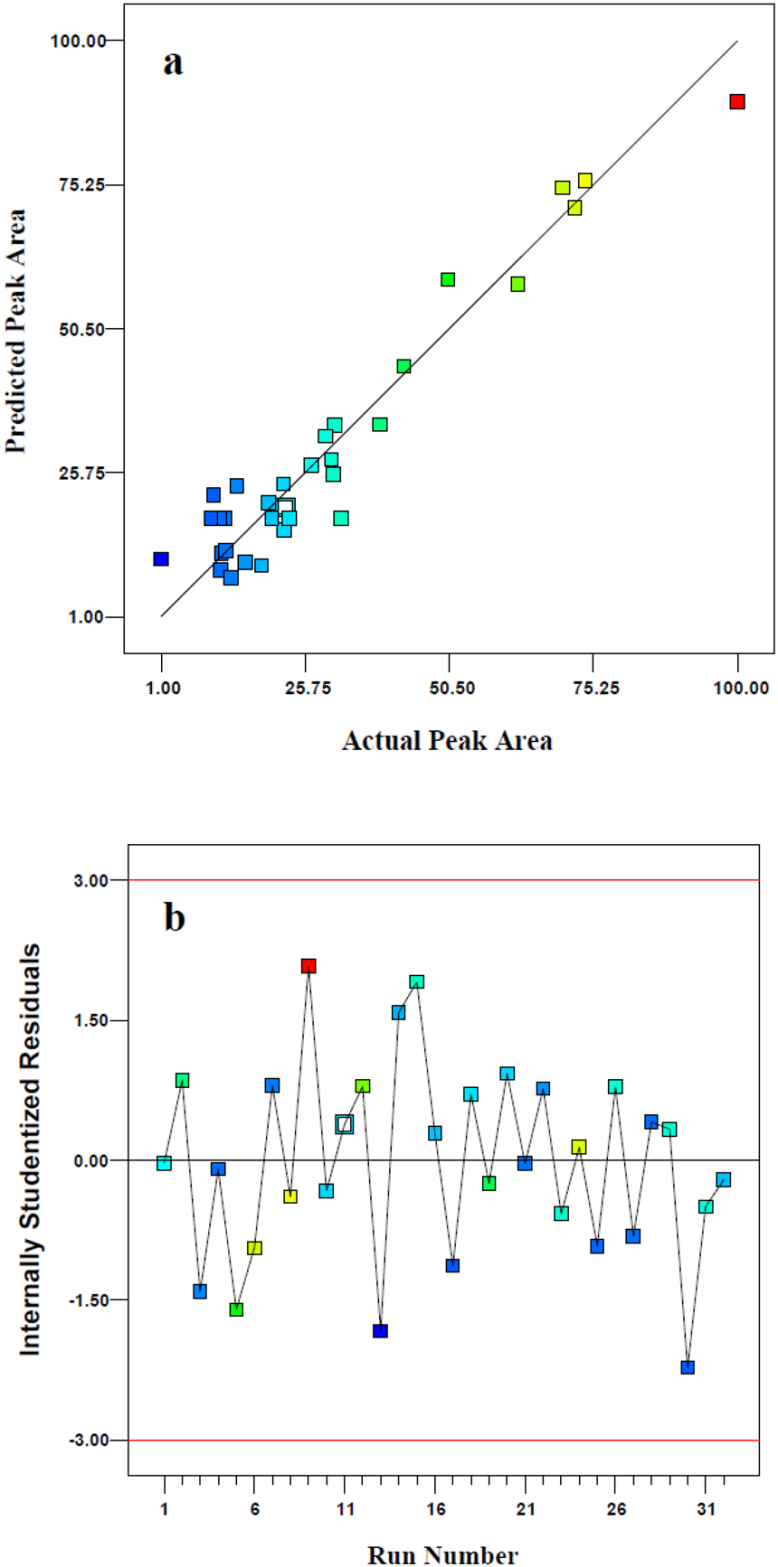
The internally studentized residuals vs. the performed runs (**a**); and the predicted value vs. actual response (**b**).

Moreover, to evaluate the overall fitness and predictive potential of the model, the squared regression coefficients, including the calibration R^2^ (R^2^_cal_), adjusted R^2^ (R^2^_adj_), and R^2^ of prediction (R^2^_pred_) were calculated^35^.As denoted in Table 2, R^2^_cal_ shows that the suggested CCD model covers 92.8% of data. Based on literature, the amount of R^2^_adj_ was bigger than 0.8, which confirms its goodness of fit^36^. Besides, the R^2^_pred_ (= 0.807) was consistent with the R^2^_adj_ (= 0.894). The closeness of R^2^_pred_ and R^2^_adj_ with a difference lower than 0.2 is an indicator for the very good prediction ability. Another statistical metrics for measuring the Signal to Noise(S/N) ratio is the adequate precision. As it is well known in literature, a ratio greater than 4 indicates an acceptable precision^36^. According to the results shown in Table 2, the value of 18.54 for adequate precision indicates a suitable S/N. All of the above-mentioned metrics are indicators of suitable correlation between the included factors and interaction terms in Eq. (2) with the peak area as the response value of the extraction recovery of t,t-MA using DLLME-SFOD. To show the goodness of fit in the propped model, the plot of predicted peak area versus the obtained experimental values is represented in Fig. 2a. One of the criteria for showing the applicability domain of a multiple linear regression model is laying the residual value (difference of actual and prediction response) between the accepted range of ± 3σ^37^. As shown in Fig. 2b, the studentized residual of the obtained model had a narrow range within ± 2σ which shows the reliability of model. On the other hand, the residual value of all the runs are scattered randomly in both side of zero line which confirms the absence of systematic error^37^.

**Table 2:** Analysis of variance (ANOVA), model statistics summery and quality of the quadratic model for the extraction of t,t-MA.

Source of Variation	Sum of square	*DF*^*a*^	*Mean square*	*F-value *^*b*^	*P-value*	*Importance*
Model	15,412.27	10	1541.23	27.23	< 0.0001	Significant
A	620.32	1	620.32	10.96	0.0033	
*B*	340.71	1	340.71	6.02	0.0230	
*C*	182.91	1	182.91	3.23	0.0866	
*D*	22.37	1	22.37	0.40	0.5363	
*E*	327.69	1	327.69	5.79	0.0254	
*AC*	904.65	1	904.65	15.98	0.0007	
*AE*	3496.50	1	3496.50	61.78	< 0.0001	
*CD*	233.92	1	233.92	4.13	0.0549	
*CE*	1547.05	1	1547.05	27.34	< 0.0001	
*E*^*2*^	7736.15	1	7736.15	136.69	< 0.0001	
Residual	1188.51	21	56.60			
Lack of Fit	810.18	16	50.64	0.67	0.7535	Not significant
Pure Error	378.33	5	75.67			
Cor Total	16,600.78	31				
**Model statistics**						
R^2^_cal_	R^2^_Adjusted_		R^2^_Pred_		Adequate precision	
0.93	0.89		0.81		18.54	

^a^Degree of freedom.

^b^Test to comparing model variance with residual variance or significance of included factors.

### Optimization of effective variables using CCD

**Figure 3:**
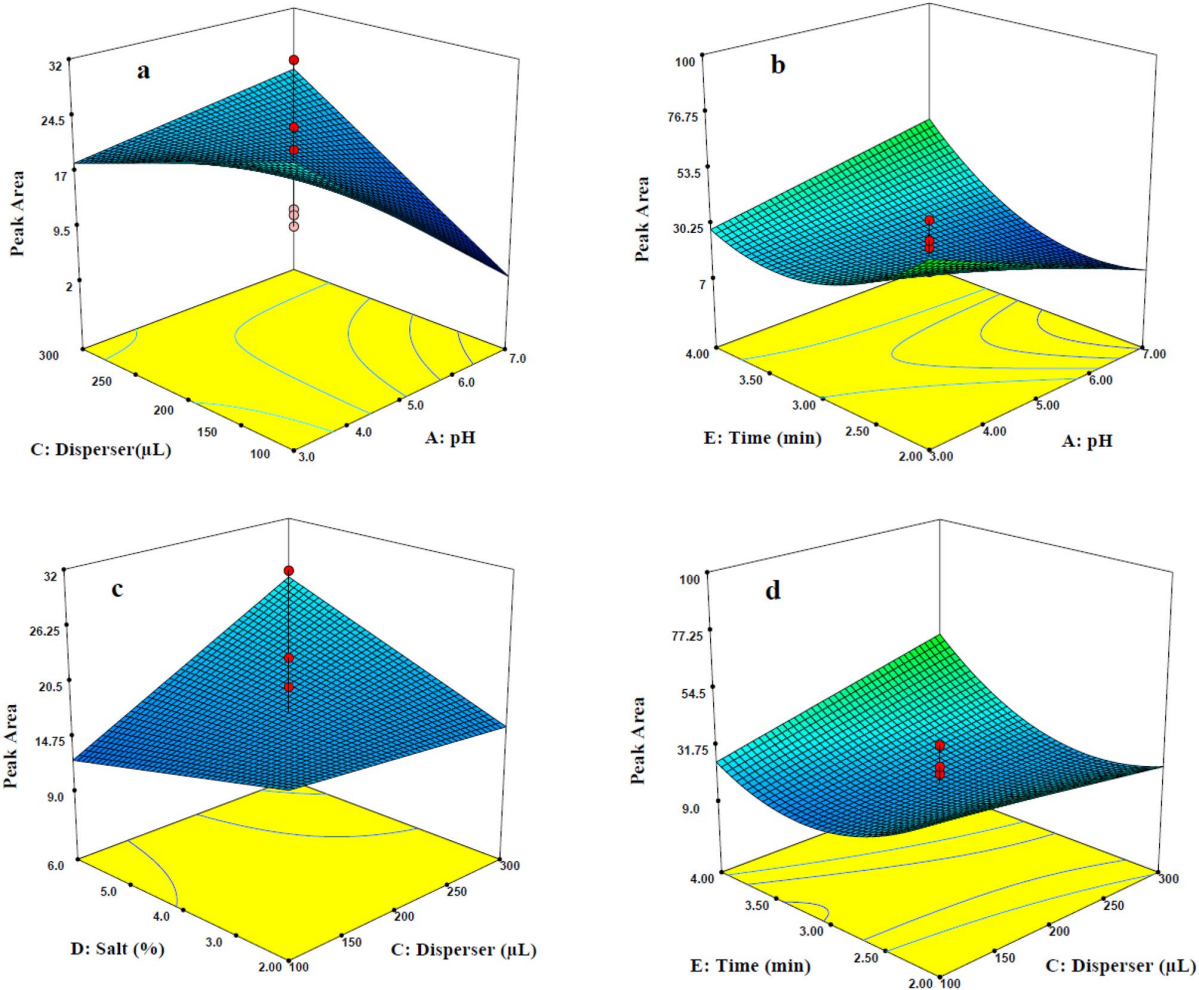
three dimensional response surface plots of between-factor interaction terms (volume of disperser-pH (**a**), time–pH (**b**), salt- volume of disperse (**c**) and time-volume of disperser (**d**) on the extraction of t,t-MA.

According to which was shown in Eq. (2), some interaction terms was entered in the multiparameter model, including between-factors-interactions (such as *AC*, *AE*, *CD* and CE) and self-interactions (E^2^). The three-dimensional (3D) response surface curves were applied to present the mixed effect of factors in between-factors-interaction terms, which can provide useful information to find optimal values of the independent parameters. These curves provide important information about maximum response and possible interactions between two independent variables. Figure 3a illustrates the simultaneous effect of pH of sample and disperser solvent volume on the response (peak area). In all extraction techniques in which analytes are basic or acidic, pH is an important parameter for extraction. To survey the influence of pH of sample solution on extraction of t,t-MA, various designed experiments were performed. As indicated in Fig. 3a, the maximal response achieved in the pH of 3.0, and the peak area increased by decreasing the pH and disperser solvent volume. At pH values greater than 3.0, the response is decreased because of the fact that the ratios of the ionic to molecular forms of the target molecule can be influenced by pH value of sample solutions^38^. Therefore, acidification of the sample is essential to achieve the highest extraction of t,t- MA. With increasing the pH, t,t-MA (pKa = 3.87) is converted to negative ionized species, which could reduce the agglomeration of the tiny droplets of 1-undecanol containing the migrated t,t-MA to create the floating phase. In order to assay the effect of the volume of disperser solvent, different examinations were designed using several volumes of methanol in the ranges of 100–300 μL. The highest response level (peak area) was obtained using 100 μL of methanol. It is because of highly solubility of 1-undecanol in methanol that generate small droplets of extractant solvent and increase the extraction output. To investigate the effect of the volume of extractant solvent, various tests were conducted using different volumes of 1-undecanol (20–60 μL). The optimization process obtained 49.0 µL of 1-undecanol as the best value of highest extraction efficiency. Therefore, this value was used to extract t,t-MA from the spiked or real samples.

The variation in extraction time of t,t-MA versus pH (Fig. 3b) shows that maximum signal of analyte was obtained after 2.0 min of extraction. The time interval between the injection of the optimum values of extraction and disperser solvents to the sample solution and centrifugation is defined as the extraction time^39^. The impact of extraction time was studied in the ranges of 2–4 min; the findings showed that high extraction efficiency was obtained at a short time. In DLLME-SFOD method, the equilibrium state is rapidly reached because of the high contact area between aqueous sample and extractant solvent and good dispersion of the analyte. Therefore, rapid and high mass transfer of the t,t-MA from aqueous media to the 1-undecanol is occurred.

As Fig. 3c indicates, the maximum extraction of t,t-MA was occurred at low concentration levels of sodium chloride and low volume of disperser solvent. In high volumes of disperser, increasing the salt can restrict the viscosity of the solution and mass transfer of the analyte. Moreover, increasing salt level and disperser solvent volume will increase the volume of floating organic phase which could decrease the enrichment factor and extraction efficiency^31,40^.

Figure 3d indicates the interaction between extraction time and disperser volume of the sample. At the constant volume of extractant solvent, by decreasing the disperser solvent volume and the extraction time the extraction of the target was increased (Fig. 3d). Enhancing the volume of disperser solvent affects the solubility of t,t-MA in the sample solution which decrease the extraction of t,t-MA. Moreover, following the generation of cloudy state, the extraction time is almost short due to the high contact area between aqueous phase and extractant solvent. This process facilitates the diffusion of target analyte into the extractant solvent with the lowest amount of disperser solvent. The use of small volume of organic solvent and also rapid extraction time are considered as the main advantages of the optimized method.

After confirming the validity of model, simplex optimization was used to find the optimum values of experimental conditions of the t,t-MA extraction procedure which were obtained as follow: pH of the sample: 3.0, the volume of extractant solvent: 49.0 µL, the volume of disperser solvent: 100 µL, salt amount: 2.0 w/v%, and extraction time: 2.0 min.

#### Matrix effects

Matrix effect was defined using the percentage of signal repression/enrichment obtained for urine samples by the proposed optimized method. In this regard, at a defined concentration level, the peak area acquired from the analysis of the fortified urine samples were compared with the relative spiked mobile phase. Based on the obtained results, the recoveries were 91–94% (n = 5). Trivial matrix effects was found when blank samples were fortified with 1.0 μg mL^−1^ t,t-MA.

### Method validation

To assay the efficiency of the optimized method, the figures of merit such as the linear dynamic ranges (LDRs), squared correlation coefficients of the analytical curve (R^2^), limit of detection (LOD), limit of quantification (LOQ), precision, and relative recovery (RR) were all examined and the obtained findings are presented in Table 3. The linearity of the optimized method was investigated by spiking seven urine specimens with increasing concentrations of t,t-MA from the LOQ to 5 μg mL^−1^. As demonstrated in Table 3, a suitable linearity was attained in the ranges of 0.02–5 μg mL with a significant correction coefficent (R^2^ of 0.997). The LOD (S/N = 3) and LOQ (S/N = 10) were 0.006 μg mL^−1^ and 0.02 μg mL^−1^, respectively. The PF was estimated using the subsequent equation:

3 $${\text{PF}} = {\text{C}}_{{{\text{ex}},{\text{final}}}} /{\text{C}}_{{{\text{aq}},{\text{initial}}}}$$

where C_ex, final_ and C_aq, initial_ are the final and initial concentration levels of metabolite in extracting and aqueous phase, respectively. Under the optimize conditions, the PF of t,t-MA was 80. Precision study, characterized in term of RSD% for reproducibility (inter-day precision) and repeatability (intra-day precision), was determined by spiking urine specimens purchased by an unexposed volunteer in the analytical lab. The proposed technique was used for measurement of the concentration of t,t-MA in urine sample taken from unexposed person. According to the results, no levels of t,t-MA was detected in the obtained urine sample. In the next step, the aliquots of the collected urine sample were transferred to different test vials and fortified with various concentrations of t,t-MA. The prepared fortified samples were analyzed by the optimized method. According to Table 3, the RSD% were below 6.5 in all concentration levels, indicating a good precision achieved by this method. The proximity of the experimental findings from a developed procedure to the real amount of the target analyte is defined as the accuracy of an analytical procedure. The application of Certified Reference Materials (CRMs) is considered as an accurate way to examine the accuracy of a procedure. Since no CRM was available for the studied analyte, the trueness of the developed method was determined by the added-found procedure. To do this, the urine samples were spiked with t,t-MA analyte at three concentration levels (0.2, 1.0 and 2.0 μg mL^−1^) and were determined in three replicates according to the optimum conditions (pH of the sample: 3.0, the volume of extractant solvent: 49.0 µL, the volume of disperser solvent: 100 µL, salt amount: 2.0 w/v%, and extraction time: 2.0 min). As shown in Table 3, a good recovery obtained with the current method in the range of 86.9–91.3%.

**Table 3:** Analytical characteristics of the method.

Correlation coefficient (r^2^)	LDR (μg mL^−1^)	LOD ( μg mL^−1^)	LOQ (μg mL^−1^)	Spiked Level (μg mL^−1^)	Accuracy	Precision (n = 3)	
					Recovery (%)	Intra-day (RSD%)	Inter-day (RSD%)
**0.997**	0.02–5	0.006	0.02	0.2	86.9	5.9	4.8
				1	90.3	6.3	4.3
				2	91.3	4.3	3.8

**Figure 4:**
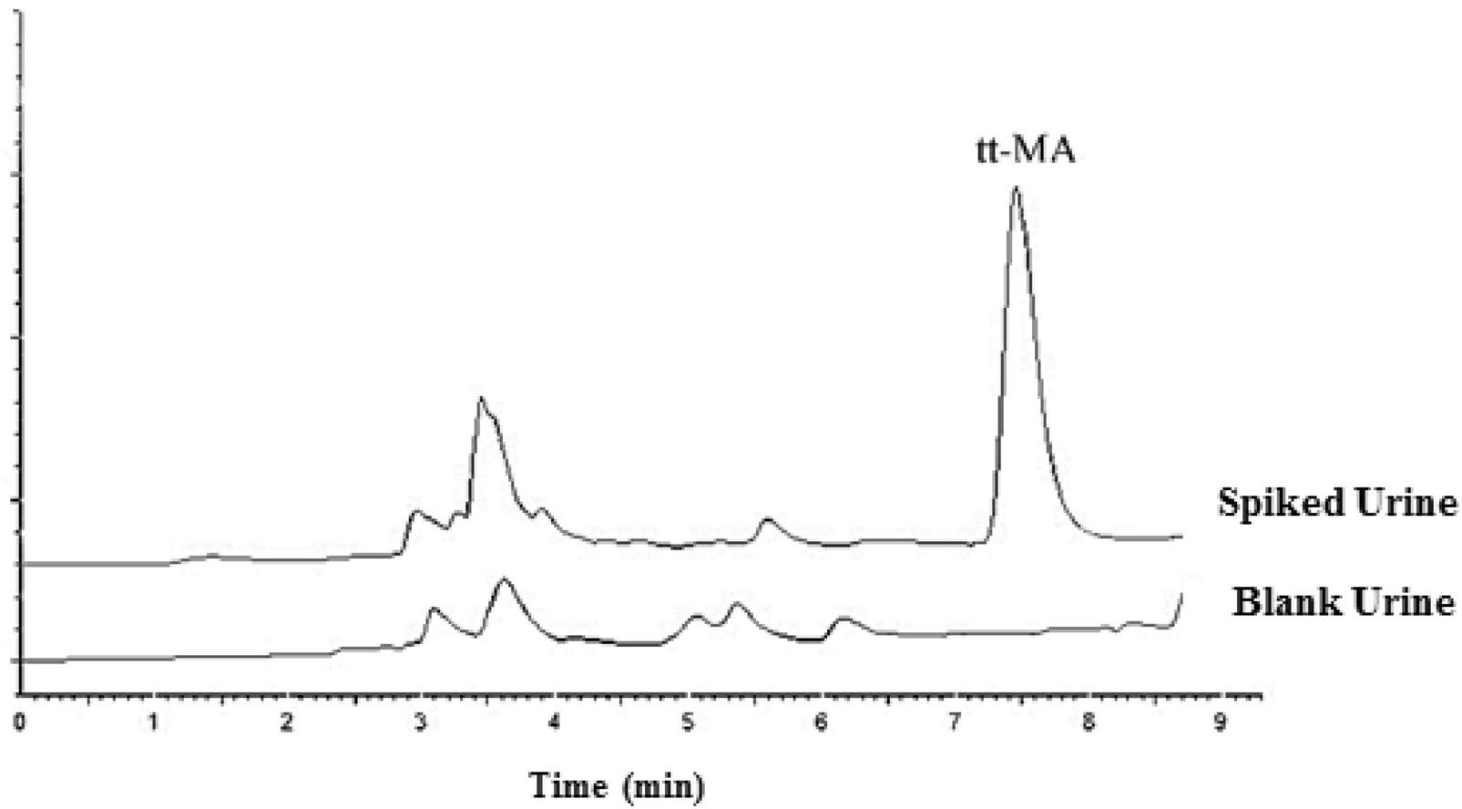
Representative HPLC chromatograms of a real sample from a non-exposed healthy man before (blank) and after spiking with t,t-MA at concentration level of 1 μg/mL. (The extraction conditions were as follow: pH of the sample: 3.0, the volume of extractant solvent: 49.0 µL, the volume of disperser solvent: 100 µL, salt amount: 2.0 w/v%, and extraction time: 2.0 min).

Figure 4 presents the chromatograms of a real urine sample collected from unexposed healthy man before and after spiking with 1.0 μg/mL of t,t-MA. As shown in Fig. 4, no chromatogram was observed at the retention time of target metabolite, indicating acceptable specificity and not require to the additional clean up step to remove interferences before HPLC analysis. As seen in Fig. 4, the analyzed real sample was free from analyte and t,t-MA could be efficiently extracted from urine samples using DLLME-SFOD technique.

### Applicability of the optimized method for urine analysis of workers who occupationally exposed to benzene

The optimized procedure was utilized for preconcentration and measurement of t,t-MA in urine samples taken from workers who occupational exposed to benzene in a petrochemical industry. Five end-shift urine specimens were collected from non-smoker employees who were occupationally exposed to benzene. All of urine samples were treated according to the optimized technique of DLLME-SFO, and analysis were performed using HPLC–UV. The results showed that t,t-MA were identified in all of taken samples. The results were expressed as urinary measured t,t-MA to creatinine (mg/g creatinine). The modification of urinary dilution was performed using urinary creatinine because the urinary level of t,t-MA is affected by urine volume. Table 4 presents the findings of the real urine analysis. On the other hand to make another estimation from the accuracy, the results were compared by the solid phase extraction technique (SPE) using SAX cartridge and HPLC–UV detection^2^, which are shown in Table 4 as well. As shown, the present technique is comparable with SPE method as a routine and well-evaluated technique in occupational laboratories.

**Table 4:** Analysis of urine samples taken from workers of a petrochemical industry using two different extraction technique: DLLME-SFOD and SPE.

Human urine samples	t,t-MA level (mg/g creatinine) MEAN ± SD, N = 3	
	DLLME-SFOD	SPE
1	1.20** ± **0.11	0.99** ± **0.08
2	1.46** ± **0.13	1.70** ± **0.80
3	1.64** ± **0.15	1.11** ± **0.12
4	0.81 ± 0.09	0.97 ± 0.06
5	0.54 ± 0.06	0.65 ± 0.05

### Comparison of the optimized method with other pretreatment methods

The analytical performance of the proposed method was compared with other previously reported techniques for the extraction and measurement of t,t-MA. As illustrated in Table 5, different solid and liquid phase extraction/ micro extraction procedures have been introduced for monitoring of t,t-MA from urine sample. As seen, the introduced method has an acceptable LOD which is comparable with other analytical techniques used for the measurement of t,t-MA. Moreover, the very low volume of the used organic extractant solvent with less toxicity in the present method are the major advantages of our suggested method. The proposed technique uses a few microliters of an organic extractant solvent without any other toxic agents such as chlorinated organic solvents. In addition, the extraction process last less than 15 min, which is more rapid than other techniques such as conventional LLE or SPE (> 30 min). Although other extraction techniques especially solid phase-based methods have a lower LOD, these techniques require proprietary instruments, expensive SPE cartridges, and complicated steps of sample preparation for the extraction of analyte. These comparisons reveal that the introduced method (DLLME-SFOD) is easy, fast, cost effective, and environmentally friendly. Moreover, 1-undecanol is an inexpensive and easily available solvent, which is appropriate for fast extraction of large-scale samples.

**Table 5:** Comparison of the SFO-DLLME method with other preconcentration techniques for monitoring of urinary t,t-MA.

Extraction technique	Organic solvents and volume	Analytical technique	Linear range (μg mL^−1^)	Extraction time (min)	LODs (μg mL^−1^)	RSD%	References
SPE	sorbic acid:50 μL HCL: 900 μL Methanol: 1 mL ammonia: 1 mL ethyl acetate: 1 mL formic acid–ethyl acetate: 800μL methanol and ortho-phosphoric acid (2:98v/v):90 μL	HPLC–UV	0.4–6.8	NR	0.005	2–7.1	^41^
LLE	HCl:1 mL Diethyl ether:10 mL ethereal diazomethane 800 μL	GC-FID	0.03–1.2	NR	0.02	9.7	^42^
MIP- DLLME	dimethyl sulphoxide (2 mL) ethanol: 7 mL ethanol/acetic acid (8:2, v/v): 4 mL pyridine:100 μL trichloroethylene: 80 μL ethyl chloroformate:100 μL	GC–MS	0.125–2	NR	0.037	5.1	^43^
MIP-MEPS	Ethanol:300 μL ethanol-acetic acid (80:20, v/v): 200μL	HPLC–UV	0.015–2	5	0.015	3.4–6.6	^44^
HFLPME	Different solutions for condition, washing and elution steps 450µL	HPLC–UV	0.005–1.2	120	0.001	2.7–7.3	^45^
IP-HF-LPME	Acetone, 1-octanol:24 μL	HPLC–UV	0.001–0.9	60	0.0001	2.7–6.1	^46^
PDLLME	Chloroform: 200 μL Tetrahydrofuran: 2000 μL	HPLC–UV	0.1–10	< 15	0.0001	6.3–14	^47^
DLLME-SFOD	Matanol:100 μL 1-undecanol:49 μL	HPLC–UV	0.02–5	< 10 min	0.006	4.3–6.3	(Present method)

*SPE* solid-phase extraction, *LLE* Liquid–liquid extraction, *DLLME* dispersive liquid–liquid micro-extraction, *MIP-DLLME* molecular imprinting polymer-Dispersive liquid liquid microextraction, *MIP-MEPS* molecular imprinting polymer–micro-extraction by packed sorbent, *HFLPME* Hallow fiber liquid phase microextraction, *IP-HF-LPM* Ion-pair-based hollow-fiber liquid-phase microextraction, *HPLC* high-performance liquid chromatography, *PDLLME* Partitioned dispersive liquid–liquid microextraction, *DLLME-SFOD* Dispersive liquid liquid microextraction-Solidified organic droplet. *NR* Not Required.

### Ethics approval

The manuscript has been approved by the ethics committee of Shiraz University of Medical Sciences (Ethical ID: IR.SUMS.REC.1398.1051). No clinical trial was performed in the study but for urine sampling as the experiments involving human participants informed consent have been obtained.

## Conclusion

In the proposed method, a novel, simple, and sensitive DLLME-SFOD method was optimized and validated for the preconcentration and trace measurement of t,t-MA by HPLC–UV in urine sample. In this study, an organic extractant solvent with an appropriate melting point was used; after solidification, it was easily collected. In comparison with other previous techniques, this method enjoys the advantages such as simplicity, low cost, low usage of organic solvent, rapidity, and environmental friendly. In this work, for the first time, DLLME-SFOD was used for the trace determination of t,t-MA in urine samples, which revealed wide linearity, satisfactory relative recovery and good precision. Especially, the LOD of DLLME-SFOD method was 0.006 μg/mL, which is lower or comparable with other previous techniques. Furthermore, the most important advantages of this method is that the extraction of t,t-MA occurs in a short time, which can be used as an efficient method in analytical labs. Because the optimized method does not require any complicated instrument and expensive material, it can be used as a simple protocol for monitoring of employees who occupationally exposed to benzene.

## Supplementary Information

Supplementary Information.
