# Supplementary material for: Solidified floating organic droplet microextraction coupled with HPLC for rapid determination of trans, trans muconic acid in benzene biomonitoring
Source: Sci Rep. 2021 Aug 3;11:15751. doi: 10.1038/s41598-021-95174-5 (PMC8333051; doi:10.1038/s41598-021-95174-5)
Supplement: Supplementary file 1 — Supplementary Information. [file 41598_2021_95174_MOESM1_ESM.pdf]

Supplementary Information

Solidified floating organic droplet microextraction coupled with HPLC for rapid determination of trans, trans muconic acid for benzene biomonitoring

Fatemeh Dehghani[1], Fariborz Omidi[2], Omidreza Heravizadeh[3], Saeed Yousefinejad[4*]

[1]*Student research committee, Shiraz University of Medical Sciences, Shiraz, Iran*

[2]*Research Center for Environmental Determinants of Health (RCEDH), Health Institute, Kermanshah University of Medical Sciences, Kermanshah, Iran*

[3] *Division of Metabolomics, Medical Institute of Bioregulation, Kyushu University, 3-1-1 Maidashi, Higashi-ku,Fukuoka 812-8582, Japan.*

[4]*Research Center for Health Sciences, Institute of Health, Department of Occupational Health and Safety Engineering, School of Health, Shiraz University of Medical Sciences, Shiraz, Iran*

***Corresponding author:** Saeed Yousefinejad

Department of Occupational Health, School of Health, Shiraz University of Medical Sciences, Shiraz, Iran

Postal code: 71645-111

E-mail: yousefisa@sums.ac.ir; yousefinejad.s@gmail.com; Fax: +98 71 37256006

https://orcid.org/0000-0001-5940-1229

Table S1. The matrix of central composite design and the obtained value of responses (peak area and normalized peak area).

Run No.	**A ***	**B ***	**C ***	**D ***	**E ***	Peak Area	Normalize Peak area
1	3	30	300	2	2	540967	26.77
2	3	30	300	6	4	780451	38.62
3	5	40	400	4	3	282986	14.00
4	7	30	300	6	2	229086	11.34
5	7	30	300	2	4	1016322	50.29
6	5	40	200	4	1	1413899	69.96
7	9	40	200	4	3	263611	13.04
8	7	50	300	6	4	1493327	73.89
9	5	40	200	4	5	2020949	100.00
10	3	50	100	6	4	444689	22.00
11	5	40	200	8	3	451916	22.36
12	3	30	100	6	2	1259351	62.31
13	7	50	100	6	2	20262	1.00
14	7	50	300	2	2	369577	18.29
15	5	40	200	4	3	644343	31.88
16	5	40	200	4	3	404593	20.02
17	5	40	200	4	3	192917	9.55
18	5	40	200	4	3	465902	23.05
19	3	50	300	6	2	864005	42.75
20	5	40	200	0	3	447202	22.13
21	5	40	0	4	3	244580	12.10
22	5	20	200	4	3	312500	15.46
23	7	50	100	2	4	621519	30.75
24	3	50	100	2	2	1456257	72.06
25	5	40	200	4	3	224611	11.11
26	5	60	200	4	3	619236	30.64
27	5	40	200	4	3	240654	11.91
28	7	30	100	2	2	226449	11.21
29	1	40	200	4	3	611359	30.25
30	3	30	100	2	4	202101	10.00
31	3	50	300	2	4	592375	29.31
32	7	30	100	6	4	393286	19.46

A: pH
B:Extractant solvent volume(µL)
C: Disperser solvent volume (µL)
D: Salt amount (w/v, %)
E: Extraction time(min)
